# Bowel disorders and its spatial trend in Manitoba, Canada

**DOI:** 10.1186/1471-2458-14-285

**Published:** 2014-03-27

**Authors:** Mahmoud Torabi

**Affiliations:** 1 Department of Community Health Sciences, University of Manitoba, 750 Bannatyne Ave., Winnipeg, Manitoba R3E 0W3, Canada

**Keywords:** Bayesian computation, Bowel disorders, Geographic epidemiology, Spatial cluster detection

## Abstract

**Background:**

Bowel disorders have destructive impacts on the patients social and mental aspects of life and can cause emotional distress. The risk of developing bowel incontinence also increases with age. The rate of incidence of inflammatory bowel disease in Manitoba, Canada, has been unusually raised. Therefore, it is important to identify trends in the incidence of bowel disorders that may suggest further epidemiological studies to identify risk factors and identify any changes in important factors.

**Methods:**

An important part of spatial epidemiology is cluster detection as it has the potential to identify possible risk factors associated with disease, which in turn may lead to further investigations into the nature of diseases. To test for potential disease clusters many methods have been proposed. The focused detection methods including the circular spatial scan statistic (CSS), flexible spatial scan statistic (FSS), and Bayesian disease mapping (BYM) are among the most popular disease detection procedures. A frequentist approach based on maximum likelihood estimation (MLE) has been recently used to identify potential focused clusters as well. The aforementioned approaches are studied by analyzing a dataset of bowel disorders in the province of Manitoba, Canada, from 2001 to 2010.

**Results:**

The CSS method identified less regions than the FSS method in the south part of the province as potential clusters. The same regions were identified by the BYM and MLE methods as being potential clusters of bowel disorders with a slightly different order of significance. Most of these regions were also detected by the CSS or FSS methods.

**Conclusions:**

Overall, we recommend using the methods BYM and MLE for cluster detection with the similar population and structure of regions as in Manitoba. The potential clusters of bowel disorders are generally located in the southern part of the province including the eastern part of the city of Winnipeg. These results may represent real increases in bowel disorders or they may be an indication of other covariates that were not adjusted for in the model used here. Further investigation is needed to examine these findings, and also to explore the cause of these increases.

## Background

Bowel disorders consist of a variety of diseases and syndromes including Crohn’s disease, ulcerative colitis, irritable bowel syndrome and bowel incontinence. Inflammatory bowel disease (IBD) includes Crohn’s disease and ulcerative colitis and is distinguished by the presence of chronic immunoinflammatory lesions in the large intestine wall (ulcerative colitis) or anywhere in the gastrointestinal tract (Crohn’s disease) [1]. These diseases are often diagnosed in early adulthood and are characterized by a relapsing and remitting course. Treatment for the recurrent episodes of abdominal pain, diarrhea and bleeding are necessary throughout the patients’ lifetime [2,3]. Since IBD is often diagnosed when the patients are still fairly young and these diseases have recurrent episodes, IBD can have a large negative affect on the patients’ quality of life [2]. Although the cause of IBD is unknown, potential risk factors include a family history of IBD (i.e., genetics), a history of smoking, age (early adulthood) and people who have been exposed to microorganisms early in life. Individuals who were breastfed as a baby are less likely to develop IBD. As well, people who have had an appendectomy prior to diagnosis are less likely to develop ulcerative colitis [4,5].

Functional gastrointestinal disorders are characterized by situations where there are recurrent symptoms, however, when examined there appears to be nothing wrong [6]. Irritable bowel syndrome (IBS) is a functional bowel disorder. IBS affects approximately 9% to 23% of the general population. The symptoms of IBS are abdominal pain or discomfort with a change in bowel function. Possible risk factors of IBS include genetics, stress, infection and a poor diet [7]. The treatment is aimed at reducing the symptoms of IBS and may be treated by dietary and lifestyle changes recommended by a doctor, pharmacotherapy, and psychosocial interventions [6,7].

Bowel incontinence is defined as the involuntary act of having a bowel movement. This disorder has destructive impacts on the patients social and mental aspects of life and can cause emotional distress [8]. The risk of developing bowel incontinence increases with age. It is estimated that between 2% and 18% of the general population and 50% of the people in nursing homes are affected by bowel incontinence [8,9].

In addition to the deterioration of the patients’ quality of life, bowel disorders including Crohn’s disease, ulcerative colitis, irritable bowel syndrome, and bowel incontinence have large negative economic impacts. An article from 1999 stated that Manitoba had unusually high rates of incidence of inflammatory bowel disease [10]. Therefore, it is important to identify trends in the incidence of bowel disorders that may suggest further epidemiological studies to identify risk factors and identify any changes in important factors. Trends may occur over a region and the primary outcome measure of our paper is to examine geographical variation in the number of people diagnosed as having a bowel disorder during 2001 to 2010 in the province of Manitoba, Canada.

A spatial cluster is a small region within the entire study area which has a high number of disease cases relative to the respective population [11]. Possible factors associated with disease may be found through the identification of disease clusters. This may lead to an improved understanding of etiology, which in turn may lead to further studies to find the link between exposures and disease interventions [12].

There are two main groups of statistical cluster detection methods, focused and non-focused (general). Focused cluster detection methods identify regions with a high number of disease occurrences in an area around a potential cause (i.e., a toxic waste site) [13,14]. Non-focused cluster detection methods are implemented in order to find regions, in general, with high number of disease [15-17]. Focused cluster detection methods include the circular spatial scan statistic (CSS) [18], flexible spatial scan statistic (FSS) [19], and Bayesian disease mapping (BYM) [15]. The Besag and Newell (BN) [20,21] test and the maximizing excess event test (MEET) [22] are general cluster detection methods. Non-focused tests are used to discover possible clusters in the study area, while focused tests are used to test the null hypothesis of no spatial cluster against the alternative hypothesis that a spatial cluster exists. Hence, the test statistics of focused tests (CSS, FSS and BYM) are designed to detect a potential cluster in a specific area of interest and the goal of non-focused tests (BN and MEET) is to capture any significant cluster in the entire study region without identifying a specific area of interest. A comparison of these methods is given in [23] with an application to childhood cancer in Alberta, Canada.

This paper is centered around the focused cluster detection methods. The non-informative Bayesian approach has become quite popular with advances in computational power. The Bayesian approach can be used as a modeling approach to identify the potential clusters. Data Cloning (DC), which was proposed by Lele et al. [24], is a computing algorithm to obtain maximum likelihood estimates (MLE) and their standard errors for general hierarchical models. Lele et al. [25] outlined a method to calculate the prediction and prediction intervals for the random effects in the class of generalised linear mixed models. The MLE approach, via DC, was then proposed to identify the possible clusters [26].

In this paper, the aforementioned focused approaches (CSS, FSS, BYM, and MLE) are used to analyze a real dataset of bowel disorders in the province of Manitoba, Canada, from 2001 to 2010.

## Methods

### Study subjects

This study was based on the Canadian Community Health Survey (CCHS) [27] from Statistics Canada. Information is gathered from the Canadian population regarding health status, health care utilization and health determinants from the cross-sectional CCHS. In order to provide reliable estimates at the health region level, the CCHS collects data from individuals aged twelve and older [27]. The number of people with bowel disorders in the province of Manitoba, Canada, from 2001 to 2010 is the focus of this study. The province of Manitoba is divided into five Regional Health Authorities which are further sub-divided into 67 Regional Health Authority Districts (RHADs). The geographic units used in our model are the RHADs and all of the data used in the study are related to these geographic boundaries. For simplicity, the RHADs are labelled 1,2,…,67. A population-based was also provided for each RHAD. Since the bowel disorder data used in the study came from a survey, appropriate weights established by Statistics Canada [27] were applied to the data, which was then aggregated over the study period from 2001 to 2010.

The province of Manitoba’s population was steady over the study period with approximately 1.15 million people in 2001 to 1.20 million people in 2010. The average population sizes varied across the regions with 920 people being the smallest population in region 38 and 91,633 people being the largest population size in region 62. The mean and median population sizes in Manitoba were 17,471 and 9,466, respectively. The total number of people with bowel disorders was 138,296 with a mean and median of 2064 and 858 people, respectively. These observations were based on the weighted results of people with bowel disorders across the 67 regions in Manitoba.

Important factors required for focused spatial detection approaches are the observed number of people with a bowel disorder and the expected number of people with a bowel disorder or the population size of each region. If the expected number of people with bowel disorders varies by different variables such as year, age, or gender, adjustments may be made. In our application, the expected number of disease cases was adjusted by year (1-10), age group ((0-5),(6-20),(21-40),(41,88),(89+)) and gender (male, female). The CSS, FSS, BYM, and MLE spatial focused cluster detection methods are outlined in the Appendix.

There are different assumptions for each of these four focused spatial cluster detection methods. The CSS and FSS approaches are distribution free, whereas, in the BYM and MLE methods it is assumed that the number of disease cases follows a Poisson distribution. As well, in the CSS and FSS approaches, the number of regions to be included in a cluster needs to be specified, however, this is not required for the BYM and MLE approaches. For the model-based cluster identification methods (BYM and MLE), if the model does not fit the data well, the result can be misleading. So, the *deviance residual*[28] should be also checked.

### Specific hypotheses

We specify the alternative hypotheses for the methods CSS, FSS, BYM, and MLE. We consider multiple alternatives that are tested separately. Further, let *RR*_*i*_ indicate the relative risk for the *i*-th region within clusters when compared with the region outside clusters; the latter has *RR*_*i*_ = 1. For example for cluster *X*, the *RR*_*i*_ is given by

$${\text{RR}}_{i}=\left\{\begin{array}{ll} 3 & i\in X\\ 1 & \text{otherwise} \end{array}\right)$$

## Results

The results of the four different cluster detection techniques when applied to a bowel disorder dataset in the province of Manitoba, Canada, from 2001 to 2010 are shown and compared in this section.

Based on the 67 regions, four different clusters were tested: (1) a case of no clusters (called A), (2) seven regions from the north part of the province (called B), (3) seven regions from south-central part of the province (called C), and (4) 12 regions which consist Winnipeg region (called D). For A, no region was specified as a potential cluster. The regions for scenarios B, C, and D are *B* = {31,33,34,36,38,40,41},*C* = {27,28,29,30,50,51,52},*D* = {56,57,58,59,60,61,62,63,64,65,66,67}, respectively.

**Figure 1:**
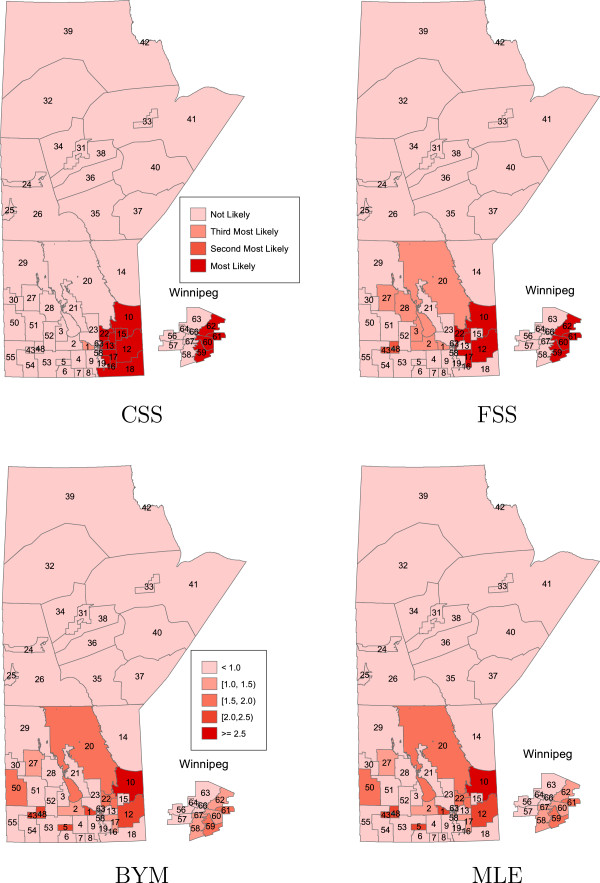
**The order of most likely clusters of bowel disorders for the CSS and FSS methods, and the spatial effects of the regional bowel disorder risks for the BYM and MLE methods; in the case of cluster A.** Major urban centre (Winnipeg region) is incorporated as inset.

**Figure 2:**
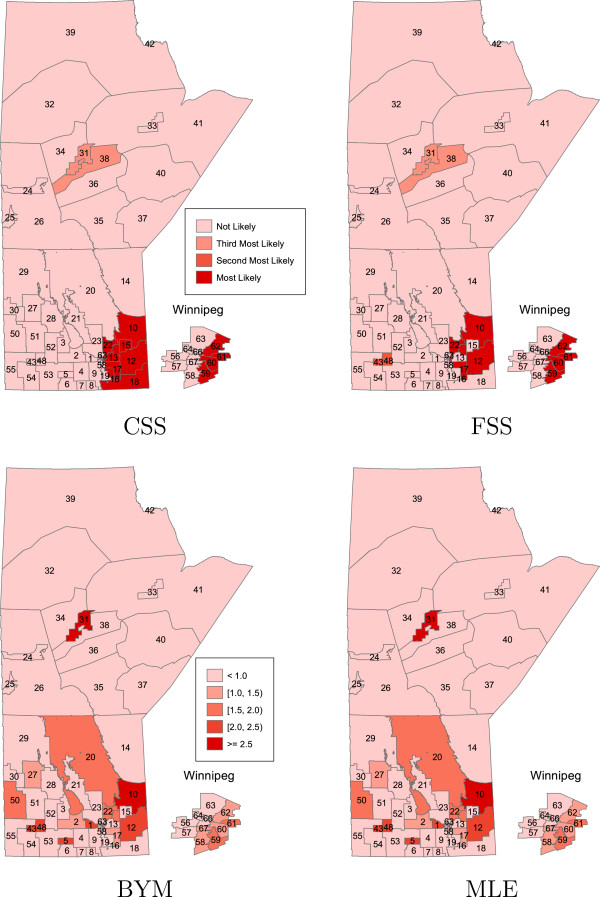
**The order of most likely clusters of bowel disorders for the CSS and FSS methods, and the spatial effects of the regional bowel disorder risks for the BYM and MLE methods; in the case of cluster B.** Major urban centre (Winnipeg region) is incorporated as inset.

**Figure 3:**
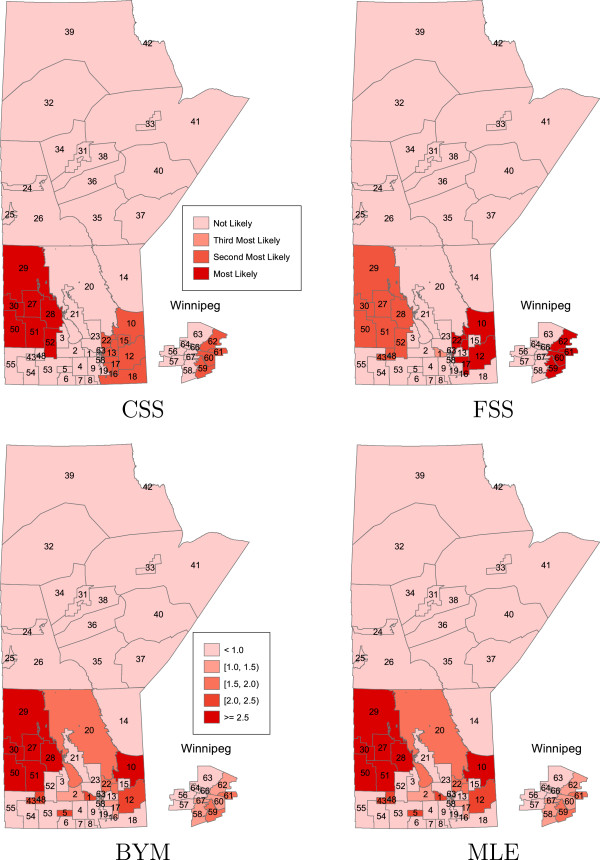
**The order of most likely clusters of bowel disorders for the CSS and FSS methods, and the spatial effects of the regional bowel disorder risks for the BYM and MLE methods; in the case of cluster C.** Major urban centre (Winnipeg region) is incorporated as inset.

**Figure 4:**
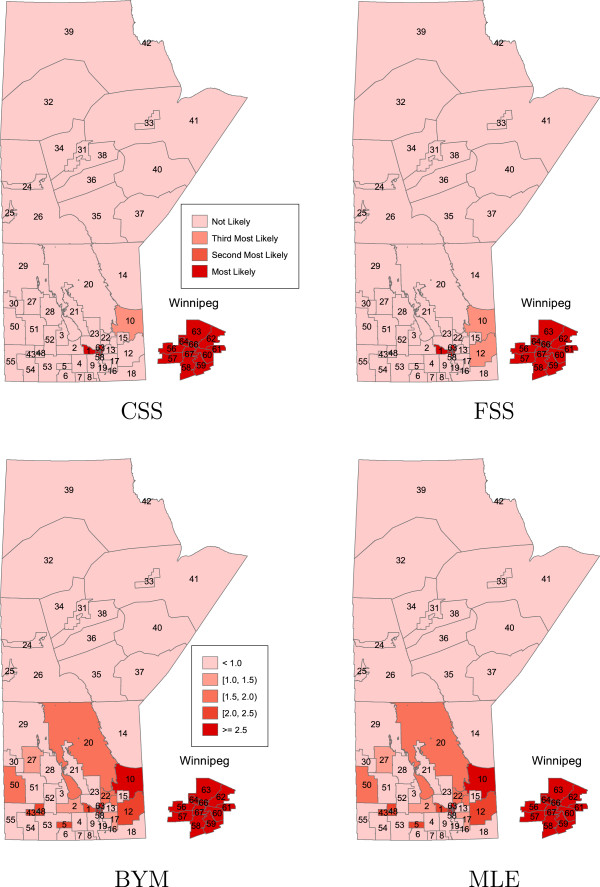
**The order of most likely clusters of bowel disorders for the CSS and FSS methods, and the spatial effects of the regional bowel disorder risks for the BYM and MLE methods; in the case of cluster D.** Major urban centre (Winnipeg region) is incorporated as inset.

In Figures 1, 2, 3, 4 the areas that are statistically significant (potential clusters) are shown for each cluster and each method separately. The summary of cluster A, no region specified as a potential cluster, is presented in Table 1. For the CSS and FSS methods, the regions that are most likely, as well as second and third most likely to be considered a cluster of disease are displayed. For the BYM and MLE approaches, each region is ranked under three criteria according to the lower limit of the credible/prediction interval. For example, for the MLE result, region 10 is most likely to be classified as a cluster of disease while region 2 is least likely to be considered as a cluster under the criteria that the lower bound of prediction intervals of RR is greater than one.

**Table 1:** The order of significant regions of the methods CSS, FSS, BYM, and MLE for cluster A

	**Method**							
**Region**			***RR > 1.0***		***RR > 1.5***		***RR > 2.0***	
	**CSS**	**FSS**	**BYM**	**MLE**	**BYM**	**MLE**	**BYM**	**MLE**
1	3	3	3	3	3	3	3	3
2	-	3	19	19	-	-	-	-
3	-	3	-	-	-	-	-	-
5	-	-	5	5	5	5	-	-
10	1	1	1	1	1	1	1	1
11	1	1	7	8	7	8	-	-
12	1	1	6	6	6	6	-	-
13	1	-	-	-	-	-	-	-
15	1	-	-	-	-	-	-	-
16	1	-	-	-	-	-	-	-
17	1	1	16	15	-	-	-	-
18	1	-	-	-	-	-	-	-
20	-	3	9	9	-	-	-	-
22	1	1	13	12	-	-	-	-
27	-	3	14	14	-	-	-	-
28	-	3	-	-	-	-	-	-
43	-	2	4	4	4	4	-	-
46	2	2	2	2	2	2	2	2
47	-	2	-	-	-	-	-	-
50	-	-	10	10	-	-	-	-
58	-	-	18	18	-	-	-	-
59	1	1	8	7	8	7	-	-
60	1	1	17	17	-	-	-	-
61	1	1	11	11	-	-	-	-
62	1	1	12	13	-	-	-	-
67	-	-	15	16	-	-	-	-

The CSS and FSS methods identified some similar regions as being potential clusters. In particular, the CSS method identified 15 regions as possible clusters while the FSS approach detected 18 regions as potential clusters of bowel disorders. The same 19 regions were identified by the BYM and MLE methods as being potential clusters of bowel disorders with a slightly different order of significance. Most of these regions were also detected by the CSS or FSS methods. Note that evaluating the criterion of the RR values from greater than 1 to 1.5 or even 2, the number of potential clusters decreases (Table 1). Based on the deviance residual plots for both methods BYM and MLE, we found that there is no serious lack of fit in the model.

For the case of cluster B, the methods BYM and MLE were only able to detect the region 31 as a potential cluster while the methods CSS and FSS also detected the region 38 as a potential cluster, noting that none of these four methods detected the other five regions (33, 34, 36, 40, 41) as a potential cluster.

For cluster C, the CSS and FSS methods detected 13 regions in addition to the cluster C as a potential cluster. The BYM and MLE methods also detected 17 regions in addition to the cluster C (except the region 52) as a potential cluster.

For cluster D, the all four methods detected the D cluster as a potential cluster. In addition to the regions in Winnipeg (cluster D), the methods BYM and MLE were also able to detect some neighbours of Winnipeg (13 regions) as potential clusters. However, the method CSS only detected three regions 1,10, and 46 as a potential cluster while the method FSS also detected the region 12 as a potential cluster in addition to regions detected by the CSS method.

## Discussion and conclusion

The four methods CSS, FSS, BYM, and MLE were studied with potential for detecting clusters with high ratio of bowel disorders in the province of Manitoba, Canada. These four methods have been extensively used in the literature and are relatively comprehensive. These methods use different approaches (semi-parametric to parametric) to test for significant clusters.

We considered four different alternative hypotheses to compare the results of different methods. In general, the CSS method identified a lower number of regions combined as a potential cluster compared to FSS method, due to non-circular shape of some regions in the province of Manitoba. It also seems that the bowel disorder cases tend to constitute the potential clusters in south-central part of the province. Note that we used four different alternative hypotheses (with low and high dense regions in terms of population) to compare these four methods, however, one can also use an extensive simulation study to compare the performance of these four methods.

The methods BYM and MLE did good jobs for dispersed population (cluster B) and also for dense population (clusters C and D) compared to the methods CSS and FSS. Also, in our study, the method FSS did a better job compared to the method CSS to detect potential clusters. Overall, we recommend using the methods BYM and MLE for cluster detection for the similar population and structure of regions as in Manitoba.

A region was identified as a potential cluster if the credible/prediction interval of the estimated relative risk was larger than one for the BYM and MLE approaches. Different decision rules may be defined where the estimated relative risk (in terms of the credible/prediction interval) would be larger or smaller than one [29]. One could also consider the exceedance probability *Pr*(*RR*_*i*_ > *b*) > *c*, where b can be 1, 2 or 3 and c might be a large value such as 0.90 [30].

Here, three important factors, age, gender and year were used to adjust the expected number of bowel disorders in the province of Manitoba. Unlike the methods CSS and FSS methods, we can extend the model (2)-(3), for both BYM and MLE methods, to include other covariates directly which may be required for some applications.

We also note that the methods have different settings and assumptions which motivate our comparison. User-chosen settings are part of all cluster tests and different choices could lead to different results. All four methods have been proposed for local clusters. Under the null hypothesis, the number of bowel disorder cases follows a Poisson distribution for the BYM and MLE methods, while the test statistic for the CSS and FSS methods has an asymptotically *χ*^2^ distribution. These features motivated us to consider these important methods and apply them to our bowel disorder cases.

As limitations of study, we assumed that our bowel disorder cases are rare cases to be able to use Poisson model in our BYM and MLE methods. We used survey data (weighted to the population level) in our study. Strengths of the study include the evaluation of multiple cluster detection methods.

The potential clusters of bowel disorders are generally located in the southern part of the province including the eastern part of the city of Winnipeg (cluster A). These results may represent real increases in bowel disorders or they may be an indication of other covariates that were not adjusted for in the model used here. Further investigation is needed to examine these findings, and also to explore the cause of these increases.

## Appendix

The CSS, FSS, BYM, and MLE spatial focused cluster detection methods are outlined below.

### Circular spatial scan statistic (CSS)

The spatial scan statistic has a variety of applications in the epidemiology field [31]. With the circular spatial scan statistic, a circular window *S* is imposed on each region. The radius of the circle ranges from zero to a pre-determined maximum distance *d* or a pre-determined maximum number of regions *J* to be considered in the cluster. The window made up of the (*j* - 1)*-th* nearest neighbours to region *i* is denoted by *S*_*i*:*j*_(*j* = 1,…,*J*). The set of all windows to be scanned by the circular spatial scan statistic is denoted by *S*_1_ = {*S*_*i*:*j*_;*i* = 1,...,*m*;*j* = 1,...,*J*}. A likelihood ratio statistic is calculated for each circle and is based on the number of observed and expected cases inside and outside the circle. The likelihood under the null and alternative hypotheses are denoted by *L*_0_ and *L*_*i*_(*i* = 1,...,*m*), respectively, where the null hypothesis states that there is no cluster in region *i* and the alternative hypothesis is there exists a cluster in region *i* based on its *j-th* nearest neighbours. The likelihood ratio statistic is given by

(1) $$\underset{i}{\text{max}}\frac{L_{i}}{L_{0}}={\left(\frac{C_{i}}{E_{i}}\right)}^{C_{i}}{\left(\frac{N-C_{i}}{N-E_{i}}\right)}^{N-C_{i}}I(C_{i}>E_{i}),$$

where the observed number of cases and expected number of cases inside a circle are denoted by *C*_*i*_ and *E*_*i*_, respectively and the observed number and expected number of cases outside a circle are denoted by (*N* - *C*_*i*_) and (*N*-*E*_*i*_), respectively. The indicator function *I*(*C*_*i*_ > *E*_*i*_) is equal to 1 when *C*_*i*_ > *E*_*i*_ and 0 otherwise. Potential clusters are identified by circles with high likelihood ratio statistics [18].

This method can be conducted using SaTScan [32] or FleXScan [33] software. The *J* is usually chosen to encompass at most 50% of the population at risk, however, we used *J* = 15, which is the FleXScan default. The region centroid had to be included in the radius of the circle in order for the region to be part of the circle.

### Flexible spatial scan statistic (FSS)

The flexible spatial scan statistic is similar to the method of the CSS except now the detected cluster is flexible in shape while still being bound to a small neighbourhood of each region. An irregularly shaped window *S* is placed on each region by the flexible scan statistic. This is done by connecting its adjacent regions. For any region *i*, the set of irregularly shaped windows of length *j*, which contains *j* connected regions including region *i*, can vary from 1 to the pre-specified maximum *J*, where *J* is the maximum length of a cluster. In order to avoid unlikely cluster shapes, the joined regions are confined to the subsets of the set of regions *i* and (*J* - 1)*-th* nearest neighbours of region *i*. The set of all windows to be scanned by the flexible spatial scan statistic is then *S*_2_ = {*S*_*i*:*j*(*k*)_;*i* = 1,…,*m*;*j* = 1,…,*J*;*k* = 1,…,*k*_*ij*_}. The size of *S*_2_ is much larger than *S*_1_ which is at most *mJ*. This is because for each region *i* the flexible scan statistic studies *J* circles plus all the sets of connected regions whose centroids are found within the *J-th* largest concentric circle, whereas the circular scan statistic considers only *J* circles for each region *i*. The likelihood ratio in (1) can be used for the flexible spatial scan statistic where the circle defined in (1) now refers to *S*_2_ rather than *S*_1_. As with the CSS method, circles with high likelihood ratio values are considered to be possible areas of disease clusters [19]. The FSS method is conducted using the FleXScan software [33], with *J* = 15, which is the FleXScan default.

### Bayesian disease mapping (BYM)

Another approach for cluster detection is a Bayesian method using Markov chain Monte Carlo (MCMC) sampling [15,16,34,35]. Bayesian disease mapping (BYM) was first used by Besag et al. [15]. Two parts are included in the model. First, the cases are assumed to follow a Poisson distribution with an area specific parameter *θ*_*i*_*E*_*i*_:

(2) $$C_{i}∼\text{Poisson}(\theta_{i}E_{i}),$$

where the observed and expected number of cases in region *i* are denoted by *C*_*i*_ and *E*_*i*_, respectively. The second part of the model comes from

(3) $$\log(\theta_{i})=\mu +\eta_{i},$$

where the relative risk (*RR*) in region *i* is given by *θ*_*i*_, *μ* represents the overall mean ratio over the entire region and the spatially correlated random effects are denoted by *η*_*i*_. The spatial random effects are found using the usual conditional autoregressive (CAR) model. However, many CAR models may be used by attaining a collection of mutually compatible conditional distributions *p*(*η*_*i*_|*η*_-*i*_),*i* = 1,…,*m* where *η*_-*i*_ = {*η*_*j*_:*j* ≠ *i*,*j* ∈ *∂*_*i*_} and *∂*_*i*_ refers to a set of neighbours for the *i-th* region [15]. The general model for the spatial effects *η*_*i*_ is

$$\begin{array}{l} \eta ={\left(\eta_{1},\ldots ,\eta_{m}\right)}^{\prime }∼N(0,\Sigma_{\eta }),\\ \Sigma_{\eta }=\sigma_{\eta }^{2}{\left(I_{m}-\lambda_{\eta }D\right)}^{-1}P, \end{array}$$

where *P* is a *m* × *m* diagonal matrix with elements *P*_*ii*_ = 1/*E*_*i*_;*D* is a *m* × *m* matrix with elements *D*_*ij*_ = (*E*_*j*_/*E*_*i*_)^1/2^ if region *i* and *j* are adjacent and *D*_*ij*_ = 0 otherwise; $\sigma_{\eta }^{2}$ is the spatial dispersion parameter; *λ*_*η*_ measures the spatial autocorrelation, *λ*_*min*_ ≤ *λ*_*η*_ ≤ *λ*_*max*_, where $\lambda_{\text{min}}^{-1}$ and $\lambda_{\text{max}}^{-1}$ are the smallest and largest eigenvalues of *P*^-1/2^*DP*^1/2^; and *I*_*m*_ is an identity matrix of dimension *m*. We refer to [36] for details of this proper CAR model. Within the Bayesian framework (MCMC) the parameters can be estimated using vague prior distributions. This produces posterior distributions for the parameters in the model given in (2)-(3) [15].

In terms of their credibility sets, when the estimated relative risk is significantly larger than one (i.e., the lower level of the credible set is larger than one) the region is considered to be a disease cluster [37]. WinBUGS software [36] was used to conduct this method and to calculate the relative risk values.

### Frequentist approach using MLE for disease mapping (MLE)

The DC approach is based on the Bayesian computational method which is used for frequentist purposes. When using the DC approach, the observations ***C*** = (*C*_1_,...,*C*_*m*_)^′^ are repeated independently by *L* different individuals. These individuals all obtain the exact same set of observations ***C*** which are denoted by ***C***^(*L*)^ = (***C***, ***C***,…, ***C***). The posterior distribution of $\alpha ={\left(\mu ,\lambda_{\eta },\sigma_{\eta }^{2}\right)}^{\prime }$ conditional on the data ***C***^(*L*)^ is then given by

(4) $$\pi_{L}\left(\alpha |C^{\left(L\right)}\right)=\frac{{\left\{L(\alpha ;C)\right\}}^{L}\pi (\alpha )}{H\left(C^{\left(L\right)}\right)},$$

where the prior distribution on the parameter space is *π*(***α***) and $H(C^{\left(L\right)})=\int {\left\{L(\alpha ;C)\right\}}^{L}\pi (\alpha )d\alpha$ is the normalizing constant. The likelihood for *L* copies of the original data is denoted by {*L*(***α***; ***C***)}^*L*^. It was shown by Lele et al. [24,25] that when *L* is large enough, *π*_*L*_(***α*** | ***C***^(*L*)^) will converge to a multivariate Normal distribution with the mean given by the MLE of the model parameters and variance-covariance matrix equal to 1/*L* times the inverse of the Fisher information matrix for the MLE. For large *L*, this distribution is almost degenerate at the MLE ***α***. In addition, an estimate of the MLE is given by the sample mean vector of the generated random numbers and *L* times their sample variance-covariance matrix is an estimate of the asymptotic variance-covariance matrix for the MLE $\hat{\alpha }$. Different tests to determine the adequate number of clones *L* were also provided by Lele et al. [25].

#### Prediction of relative risk

From a frequentist point of view, the prediction of the relative risk (random effects) is usually difficult. When ***α*** is estimated using the data, one approach is to use $\pi (R=r|C,\hat{\alpha })$ where ***R*** = (*RR*_1_,…,*RR*_*m*_)^′^, however, this method does not take into account the variability introduced by the model parameters estimate. In order to account for the variation of the estimator, one method that has been proposed and discussed in the literature [25,38] is to use the density:

(5) $$\pi (r|C)=\frac{\int f\left(C|r,\alpha_{1})g(r|\alpha_{2})\phi (\alpha ,\hat{\alpha },I^{-1}(\hat{\alpha })\right)d\alpha }{H(\text{C})},$$

where $\alpha_{1}=\mu ,\alpha_{2}={\left(\lambda_{\eta },\sigma_{\eta }^{2}\right)}^{\prime },f(\cdot )$ and *g*(·) are Poisson and Normal distributions, *ϕ*(.,*ξ*,Σ) denotes a multivariate Normal density with mean *ξ* and variance-covariance Σ and H(C)=∫L(α;C)π(α)dα is the normalizing constant. Using the density given in equation (5) and MCMC sampling, the prediction of the *r* is found. Similar to the BYM method, a region where the estimated relative risk (in terms of their prediction interval) is significantly larger than one is considered to be a disease cluster. In order to calculate the relative risk values, the dclone package [39] is used in the R software [40].

## Competing interests

The author declares that he has no competing interests.

## Pre-publication history

The pre-publication history for this paper can be accessed here:

http://www.biomedcentral.com/1471-2458/14/285/prepub
